# Outpatient treatment of alcohol use disorders among subjects 60+ years: design of a randomized clinical trial conducted in three countries (Elderly Study)

**DOI:** 10.1186/s12888-015-0672-x

**Published:** 2015-11-14

**Authors:** Kjeld Andersen, Michael P. Bogenschutz, Gerhard Bühringer, Silke Behrendt, Randi Bilberg, Barbara Braun, Claus Thorn Ekstrøm, Alyssa Forcehimes, Christine Lizarraga, Theresa B. Moyers, Anette Søgaard Nielsen

**Affiliations:** Institute of Clinical Research, Unit of Clinical Alcohol Research (UCAR), University of Southern Denmark, Odense, Denmark; OPEN, Odense Patient data Explorative Network, Odense University Hospital, Odense, Denmark; Department of Psychiatry – Odense, Sdr. Boulevard 29, 5000 Odense C, Denmark; Department of Psychiatry, New York University, School of Medicine, New York, USA; Institute of Clinical Psychology and Psychotherapy, Addiction Research Unit, Technische Universität Dresden, Dresden, Germany; IFT Institut für Therapieforschung, Munich, Germany; Biostatistics, Department of Public Health, University of Copenhagen, Copenhagen, Denmark; Department of Psychiatry and Behavioral Sciences, University of New Mexico, Albuquerque, USA; Clinical Trials Network SW Node, UNM Center on Alcoholism, Substance Abuse and Addictions, Albuquerque, USA; Department of Psychology, University of New Mexico, Albuquerque, USA

**Keywords:** Alcohol use disorder, 60+ years, Rrandomized clinical trial, Motivational Enhancement Therapy, Community reinforcement approach

## Abstract

**Background:**

The proportion of 60+ years with excessive alcohol intake varies in western countries between 6–16 % among men and 2–7 % among women. Specific events related to aging (e.g. loss of job, physical and mental capacity, or spouse) may contribute to onset or continuation of alcohol use disorders (AUD). We present the rationale and design of a multisite, multinational AUD treatment study for subjects aged 60+ years.

**Methods/Design:**

1,000 subjects seeking treatment for AUD according to DSM-5 in outpatient clinics in Denmark, Germany, and New Mexico (USA) are invited to participate in a RCT. Participants are randomly assigned to four sessions of Motivational Enhancement Treatment (MET) or to MET plus an add-on with eight sessions based on the Community Reinforcement Approach (CRA), which include a new module targeting specific problems of older adults. A series of assessment instruments is applied, including the Form-90, Alcohol Dependence Scale, Penn Alcohol Craving Scale, Brief Symptom Inventory and WHO Quality of Life. Enrolment will be completed by April 2016 and data collection by April 2017.

The primary outcome is the proportion in each group who are abstinent or have a controlled use of alcohol six months after treatment initiation. Controlled use is defined as maximum blood alcohol content not exceeding 0.05 % during the last month. Total abstinence is a secondary outcome, together with quality of life andcompliance with treatment.

**Discussion:**

The study will provide new knowledge about brief treatment of AUD for older subjects. As the treatment is manualized and applied in routine treatment facilities, barriers for implementation in the health care system are relatively low. Finally, as the study is being conducted in three different countries it will also provide significant insight into the possible interaction of service system differences and related patient characteristics in predictionof treatment outcome.

**Trial registration:**

Clinical Trials.gov NCT02084173, March 7, 2014.

## Introduction

For several decades the prevention of adolescent high-risk alcohol use and the treatment of middle aged patients with alcohol use disorders (AUD) have been major public health targets. Less attention has been paid to the development of AUD treatment programs that are tailored to specific treatment needs of older adults (60+ years) with AUD. We report on the rationale and design of a multi-centre treatment study conducted in Denmark, Germany and USA for this age group.

## Background

### Prevalence

The proportion of older adults with problematic alcohol consumption is of considerable size. In Denmark the share of men aged 65 years and older drinking more than the official Danish “high-risk” limit of 21 standard drinks (1 standard drink = 12 g pure alcohol) per week has declined from 13.8 % in 2010 to 11.7 % in 2013, with a similar decline for women drinking above the “high-risk” limit of 14 standard drinks per week from 8.2 % to 6.9 % [1]. However, proportions are high, and because of the increasing number of people reaching a higher age the absolute number is also increasing. In Germany the percentage of men aged 60–64 years drinking at hazardous level or above (>60 g pure alcohol per day) has been reported to be 6.2 % and among women in the same age group the percentage was 1.6 % (>40 g pure alcohol per day) [2]. Among older adults in residential care facilities 14 % have a misuse of alcohol [3], and among patients aged 75 years and older in general practice 6.5 % have at-risk alcohol consumption [4]. In the USA among persons aged 65 and older 16 % of men and 4 % of women reported an unhealthy drinking pattern (monthly use exceeding 30 drinks or heavy episodic drinking of four or more drinks in any single day during a typical month in the previous year) [5]. In the National Survey on Drug Use and Health 6.7 % of 65 years and older persons reported alcohol abuse or alcohol dependence according to DSM-IV, and among past-year alcohol users the proportion was 15.4 % [6]. In this age group 13 % of men and 8 % of women showed at-risk drinking, and 14 % of men and 3 % of women binge drinking [7].

### Age-related risk- factors and drinking motives

Older adults with a high level of alcohol consumption constitute a heterogeneous group with different histories of alcohol consumption. Some have started drinking excessively early in life and have survived into late adulthood continuing their level of alcohol consumption or experiencing periods of recovery and exacerbation; some have started drinking excessively during middle life, continuing their alcohol consumption which may become more problematic with increasing age; and some have started drinking excessively late in life. To our knowledge there are no data on the distribution of these different groups within older adults. Different causes for late-onset drinking have been put forward: difficulty in coping with loneliness, deteriorating social function, retirement, depression, sadness, bereavement, hopelessness, and others [8, 9]. Some may cope with losses and loneliness by drinking [10].

### Age-related negative alcohol use consequences

Light alcohol consumption has consistently been reported to have a protective effect on cardiovascular illness as well as on mortality, including all-cause mortality [11–13]. Alcohol may improve self-esteem, provide relaxation, and is often, but certainly not always, consumed socially [14]. However, AUD-related negative consequences clearly outweigh these beneficial effects, and the threshold for harmful effects becomes lower as age increases. The psychosocial consequences of AUD among the older adults may not be as severe as in younger subjects, as average severity of AUD is generally lower compared to younger persons, and therefore behaviour is less affected by the alcohol consumption [4, 15]. Excessive alcohol intake in the older adults may increase the risk of a number of conditions and diseases, including hypertension [16, 17], hemorrhagic stroke [12], diabetes mellitus [18], alcoholic liver disease and other diseases in the gastrointestinal system, including gastritis and ulcers with increased risk of bleeding, infectious diseases [19], pulmonary diseases [20], and falls with fractures as a consequence [21]. There is clear evidence that in a dose-dependent pattern alcohol increases the risk of cancer in many organs, including oropharynx, larynx, oesophagus, liver, colon-rectum, and breast [22]. In addition, older adults may have physical and mental co-morbidities that are treated with various medications, introducing risk of harmful interactions between alcohol and medication [23]. Further on, current data suggests that AUD is associated with an increased risk of depression, [24]. Suicide is closely linked to depression and alcohol use. AUD may predispose to suicidal behaviour through the increased risk of depression and promotion of adverse life events [25]. Excessive alcohol intake may be associated with increased risk of cognitive decline and dementia [26], although data are conflicting.

Other studies on treatment of AUD have included older adults, but the treatments have not been targeting the older adults and rarely data specifically on this age group are reported. As the western societies age rapidly in the coming years older adults may be an increasing challenge to treat. Given this background, we conduct a randomized controlled clinical trial for subjects aged 60+ with DSM-5 AUD, with one standard and one age-adapted brief intervention.

### Objectives

As an overall aim we strive to provide better treatment for older people with AUD. As first objective we will examine the effects of standard Motivational Enhancement Therapy (MET), a very brief treatment based on motivational interviewing. A Cochrane Review concluded that MET was clearly more effective than no treatment [27]. Since older individuals with AUD tend to be psychosocially less affected by alcohol use, brief treatment is seen at least as appropriate as it is for younger persons with alcohol problems [28]. Second, in order to specifically address the needs of older adults, we will examine whether an add-on to standard MET will produce greater improvement compared to MET alone. This add-on is The Community Reinforcement Approach (CRA) [29]. The CRA approach is based on the belief that the surrounding environment can play a powerful role in encouraging or discouraging alcohol use. Consequently, it utilizes social, recreational, familial, and vocational reinforcers to assist people in the recovery process. The underlying perspective of CRA is relatively simple: In order for a person to give up a significant reinforcer such as alcohol use, the alternative needs to be more rewarding. Because old age is a period of transitions with risk of or certainty of losses, for example retirement and loss of friends and family members, we have developed an additional module in CRA targeting the specific needs older adults may encounter. Whether or not drinking is a problem, the loss of sources of positive reinforcement may be a significant life transition as people age, and a significant decrease in positive reinforcement is a common precipitant of problem drinking [30]. Thirdly, we analyse possible culture-related differences of alcohol-related care, patient characteristics and treatment outcome in the three States participating, Denmark, Germany, and the USA. To our knowledge, this is the first study of its kind: A study specifically targeting the treatment of older adults (60+ years) with alcohol problems and including 3 countries across the Atlantic Ocean.

## Methods

### Design and settings

The study is conducted as a multicentre, multinational single-blind randomized controlled study, with outpatient alcohol treatment facilities in Denmark (3 sites), Germany (10 sites), and USA (New Mexico) (1 site). We started in spring 2014 and enrolment is expected to finish by April 2016. So far (August 2015) nearly 550 participants have been enrolled.

In Denmark the municipalities offer alcohol treatment in outpatient facilities. Treatment is free and is given to all persons contacting the outpatient clinics. Patients can be treated without a referral but can approach the clinics directly. In Germany treatment is offered by a specialized addiction care system. Counseling and treatment is free: the facilities receive city and State funds for outpatient services. In New Mexico and Germany patients over 60 years do not frequently seek treatment for addiction in specialty care settings. However, in New Mexico, they often see primary care providers, making primary care settings particularly promising for the detection and treatment of substance use disorders in older people in the USA. Therefore, in Albuquerque the study is implemented in a primary care clinic. In Germany, in addition to outpatient counselling centers that belong to the addiction care system, treatment is offered at the research institution (Technische Universitaet Dresden) or an outpatient psychotherapy clinic (Munich). In all three countries staff education is high and specialized training is mandatory. Finally, in all three countries, detoxification, if necessary, is offered before treatment.

### Subjects

Based on a power calculation (see 2.6), we aim to recruit 1,000 patients, 500 in each of the two treatment arms. Recruitment is primarily based on self-referral, including referrals from general practice, hospitals etc., to the treatment facilities. All patients aged 60 years or older who ask for treatment are informed that this project is being conducted and are recruiting participants. If a person up front is not interested in the study this person receives standard treatment according to local guidelines. If the person is interested, a session with a research assistant is set up. The person (and family members or significantly others if the person wants support or discussing the implications of participating before deciding) receives oral and written for information about the study. There is no time limit for potential participants to decide whether to participate or not, and the potential participant may return to the research group with questions before deciding to participate or not. No project procedures are conducted before a signed informed consent, which may be revoked at any time if the participant regrets participation. We strive for ecological validity and only exclude patients who are unable to participate in the therapy or are suffering from severe conditions judged to threaten their safety or the validity of the study. *Inclusion criteria:* (1) Older adults (≥60 years) with an (2) alcohol use disorder according to DSM-5 [31] and (3) before participation pass 8 out of 10 questions in a comprehension quiz pertaining to the implications of participating in the study, for example knowledge about voluntary participation and that any acceptance of participation can be withdrawn at any time. This custom-made quiz is conducted after informed consent, and is in a multiple-choice format. *Exclusion criteria:* (1) Current psychotic symptoms (2) Severe depression at the time of screening. (3) Manic episode in the last three months prior to screening or lifetime diagnosis of bipolar disorder. (4) Suicidal thoughts/behaviour at the time of screening. (5) Use of illicit opioids and/or illicit stimulants (*all other forms of medication is allowed, including opioids by prescription*). (6) Participating or having participated in other alcohol treatment programs in the last 30 days at the time of screening. Pure detoxifications are not exclusionary. (7) Patients with legally authorized representatives.

### Interventions

#### Brief MET treatment (Standard treatment)

Consists of 4 sessions, 1 per week. The intervention is based on the initial phases of the Combined Behavioral Intervention (CBI) from Project COMBINE [32, 33], and includes motivational interviewing, a functional analysis and planning for change as appropriate. In this condition, the change plan focuses exclusively on self-directed activities both during the treatment and after the 4 sessions are completed. A concerned significant other (CSO) may participate in the last session.

#### Extended treatment

Consists of 4 initial sessions similar to those in the Brief MET Treatment, except that the change plan is explicitly designed to include therapeutic modules to be completed collaboratively with the therapist. Similarly to the Brief MET Treatment, a CSO can be included in session 4. After the first 4 sessions, up to 8 additional sessions can be used by the therapist and client to implement the change plan with 1 session per week. This is accomplished through modules addressing specific areas pertaining to older age alcohol use disorders, which are together called CRA for Seniors (CRAS-S). CRAS-S consists of 5 independent modules: 1) Coping with Craving, Urges, and Social Pressure to Drink; 2) Coping with Aging; 3) Mood Management Training; 4) Building a Sober Network; and 5) Social and Recreational Counselling. These modules are based on the manual and procedures for the Combined Behavioural Intervention (CBI) as developed and tested in the COMBINE trial. Unique to this intervention, a module to manage the challenges of aging has been developed. This module, Coping with Aging, addresses older adults’ needs and problems with strategies for coping with aging and attendant losses. Formulated to encompass the 5 domains from the Happiness Form, the module on aging is adapted to the style and procedures of CRA (Table 1). Based on the change plan, the relevant topics/modules for treatment are chosen by participant and therapist. Thus the manual consists of standardized modules with detailed descriptions of the content and procedures for each module, but flexibility to allow the therapist and client to choose which modules are relevant for patient’s problem profile. The selection and order of modules and how many sessions devoted to each module is planned collaboratively, and sessions typically include more than one module at a time.

**Table 1:** Personal happiness form, adapted from the combine study [33]

Personal happiness form															
	How happy or satisfied are you with each of these areas of your life? (circle only one number for each item)											If unsatisfied, (= < 5), what is the reason? Please mark more than one if appropriate*			
Life areas	Completely dissatisfied		Somewhat dissatisfied		Somewhat satisfied		Mostly satisfied		Completely satisfied		Doesn’t apply (IR)	Loss	Loneliness	Loss of ability due to aging	Something else, please note
Growing old*	1	2	3	4	5	6	7	8	9	10	IR	0 = no	0 = no	0 = no	
												1 = yes	1 = yes	1 = yes	
												9 = irr.	9 = irr.	9 = irr.	
Inter personal relations															
Relationship with partner/spouse	1	2	3	4	5	6	7	8	9	10	IR	0 = no	0 = no	0 = no	
												1 = yes	1 = yes	1 = yes	
												9 = irr.	9 = irr.	9 = irr.	
Friends and social life	1	2	3	4	5	6	7	8	9	10	IR	0 = no	0 = no	0 = no	
												1 = yes	1 = yes	1 = yes	
												9 = irr.	9 = irr.	9 = irr.	
Family relationships	1	2	3	4	5	6	7	8	9	10	IR	0 = no	0 = no	0 = no	
												1 = yes	1 = yes	1 = yes	
												9 = irr.	9 = irr.	9 = irr.	
Love and affection	1	2	3	4	5	6	7	8	9	10	IR	0 = no	0 = no	0 = no	
												1 = yes	1 = yes	1 = yes	
												9 = irr.	9 = irr.	9 = irr.	
Sex life	1	2	3	4	5	6	7	8	9	10	IR	0 = no	0 = no	0 = no	
												1 = yes	1 = yes	1 = yes	
												9 = irr.	9 = irr.	9 = irr.	
Social life															
Leisure time and fun	1	2	3	4	5	6	7	8	9	10	IR	0 = no	0 = no	0 = no	
												1 = yes	1 = yes	1 = yes	
												9 = irr.	9 = irr.	9 = irr.	
Giving/caring for others	1	2	3	4	5	6	7	8	9	10	IR	0 = no	0 = no	0 = no	
												1 = yes	1 = yes	1 = yes	
												9 = irr.	9 = irr.	9 = irr.	
Personal safety, security	1	2	3	4	5	6	7	8	9	10	IR	0 = no	0 = no	0 = no	
												1 = yes	1 = yes	1 = yes	
												9 = irr.	9 = irr.	9 = irr.	
Life circumstances															
Job/work/pension	1	2	3	4	5	6	7	8	9	10	IR	0 = no	0 = no	0 = no	
												1 = yes	1 = yes	1 = yes	
												9 = irr.	9 = irr.	9 = irr.	
Where I live	1	2	3	4	5	6	7	8	9	10	IR	0 = no	0 = no	0 = no	
												1 = yes	1 = yes	1 = yes	
												9 = irr.	9 = irr.	9 = irr.	
Money, financial security	1	2	3	4	5	6	7	8	9	10	IR	0 = no	0 = no	0 = no	
												1 = yes	1 = yes	1 = yes	
												9 = irr.	9 = irr.	9 = irr.	
Physical health															
Physical health	1	2	3	4	5	6	7	8	9	10	IR	0 = no	0 = no	0 = no	
												1 = yes	1 = yes	1 = yes	
												9 = irr.	9 = irr.	9 = irr.	
Physical activities, exercise	1	2	3	4	5	6	7	8	9	10	IR	0 = no	0 = no	0 = no	
												1 = yes	1 = yes	1 = yes	
												9 = irr.	9 = irr.	9 = irr.	
Daily routine, the small tasks during the day	1	2	3	4	5	6	7	8	9	10	IR	0 = no	0 = no	0 = no	
												1 = yes	1 = yes	1 = yes	
												9 = irr.	9 = irr.	9 = irr.	
Eating, weight	1	2	3	4	5	6	7	8	9	10	IR	0 = no	0 = no	0 = no	
												1 = yes	1 = yes	1 = yes	
												9 = irr.	9 = irr.	9 = irr.	
Psychological health															
Mood and self-esteem	1	2	3	4	5	6	7	8	9	10	IR	0 = no	0 = no	0 = no	
												1 = yes	1 = yes	1 = yes	
												9 = irr.	9 = irr.	9 = irr.	
Stress and anxiety	1	2	3	4	5	6	7	8	9	10	IR	0 = no	0 = no	0 = no	
												1 = yes	1 = yes	1 = yes	
												9 = irr.	9 = irr.	9 = irr.	
Anger and arguments	1	2	3	4	5	6	7	8	9	10	IR	0 = no	0 = no	0 = no	
												1 = yes	1 = yes	1 = yes	
												9 = irr.	9 = irr.	9 = irr.	
Spirituality	1	2	3	4	5	6	7	8	9	10	IR	0 = no	0 = no	0 = no	
												1 = yes	1 = yes	1 = yes	
												9 = irr.	9 = irr.	9 = irr.	
Memory	1	2	3	4	5	6	7	8	9	10	IR	0 = no	0 = no	0 = no	
												1 = yes	1 = yes	1 = yes	
												9 = irr.	9 = irr.	9 = irr.	
Mental ability	1	2	3	4	5	6	7	8	9	10	IR	0 = no	0 = no	0 = no	
												1 = yes	1 = yes	1 = yes	
												9 = irr.	9 = irr.	9 = irr.	

*Added a question about aging in general and if dissatisfaction in any domain is reported, then is it because of factors related to aging

### Instruments and measurement

Patients will be assessed at screening, at baseline, at the end of MET treatment (4 weeks after baseline), at the end of MET + CRA treatment (12 weeks after baseline), at six months after baseline (the time of primary outcome), and at 12 months after baseline (See Table 2).

**Table 2:** The evaluation instruments

Assessment	Purpose/Content	BL	Wk 4	Wk 12	Wk 26	Wk 52
Alcohol consumption, dependence, and criteria for AUD						
Form 90-AI/F [38]	Primary outcome: daily alcohol use	•	•	•	•	•
Alcohol Dependence Scale [39, 47]	Alcohol Dependence	•			•	•
Drinker Inventory of Consequences [48]	Adverse consequences of drinking	•	•	•	•	•
Mini-International Neuro-psychiatric interview [34]	Module J: Assess whether the patient continuously fulfil the DSM-5 criteria for AUD	•	•	•	•	•
Motivation						
Importance, Confidence, Readiness [49] (3 Likert scales)	Change in motivation	•	•	•	•	•
Craving						
Penn Alcohol Craving Scale [40]	Changes in craving	•	•	•	•	•
Alcohol Abstinence Self-Efficacy Scale T/C [41]	Changes in self-efficacy	•	•	•	•	•
Psychological and Psychiatric assessment						
Brief Symptom Inventory 18 [36]	Changes in anxiety, depression etc.	•	•	•	•	•
Quality of life, domains with problems related to aging						
WHOQOL-BREF [42]	Changes in quality of life	•	•	•	•	•
Personal Happiness Form [33]	Identifying and assessing changes in interpersonal relations (family, friends, etc), life circumstances (housing, work/retirement, etc.), physical and psychological health	•	•	•	•	•
Therapy Compliance and Treatment Satisfaction						
Questionnaire on “Goal of treatment”	Questions on what the patients wants from treatment	•				
Session Record Form^a^	Quality control	•	•	•		
Treatment Satisfaction Form	Patients’ evaluation of treatment		•	•	•	•
Hair sample of 10 % of patients	Measuring alcohol to assess alcohol consumption the last 30 days				•	

BL: Baseline

Wk: week

^a^Completed after every session during MET- and CRA-treatment, week 1–12

*Screening* includes Modules J (alcohol), A (depression), D (manic episode), and L (psychosis) of the Mini-International Neuro-psychiatric Interview (M.I.N.I.) (version 5.0.0) to assess inclusion and exclusion criteria [34]. To comply with the DSM-5 criteria for alcohol use disorder two questions on craving have been added in module J of the M.I.N.I. [35].

*Baseline assessment* includes the remaining modules in the M.I.N.I. and the BSI (Brief Symptom Inventory) [36] in order to assess the current psychopathology burden of the patient. The Charlson Comorbidity Index is used to assess the patient’s physical status, including a self-report of the patient’s current medication [37]. In addition to this, data on demographics are collected: highest level of education, current work situation (type of job, retired, unemployed, etc.), marital status, family and friends, type of housing, and hobbies and interests, including out-of-house activities. Further, to examine the patient’s attitude towards treatment in general, the patient is asked about what he or she wants from treatment (e.g. abstinence or controlled consumption). Weight and height is also collected and used for the Blood Alcohol Content (BAC) calculations.

Across *baseline* and *follow-up* assessments several aspects of alcohol consumption are measured (see Table 2): alcohol consumption (Form-90) [38], severity of dependence (Alcohol Dependency Scale) [39], craving (Penn Alcohol Craving Scale [40] and Alcohol Abstinence Self-Efficacy Scale [41]), and motivation for change and treatment, the latter including therapy compliance and treatment satisfaction (custom-made questionnaires). Further, changes in quality of life (WHOQOL-BREF) [42] and problems related to aging, psychological and psychiatric problems are assessed (Personal Happiness Form) [33].

At *6 months follow-up* hair-samples are collected from all participants.

### Outcome measures and hypotheses

#### Primary outcome

- Proportion of patients with abstinence or controlled use (maximum alcohol resulting in an estimated BAC ≤ 0.05 %) in the last 30 days at 6 months after start of treatment.

Although “controlled use” is included as a positive outcome in the primary outcome analysis, secondary analyses will examine total abstinence as a treatment outcome. In the program, the therapist as a treatment goal recommends abstinence. However, participants who are unwilling to become abstinent are supported in achieving a reduction of their alcohol use.

#### A priori hypotheses

1. Patients randomly assigned to a brief outpatient behavior therapy program (MET; Treatment Group 1) will show a significant improvement of their drinking pattern between onset, end of treatment and 6- and 12-month follow-up.
2. Patients randomly assigned to a more intensive outpatient behavior therapy (MET plus CRA; treatment group 2) will show a significant *greater* improvement of their drinking pattern between onset, end of treatment and 6- and 12-month follow-up than treatment group 1. A clinically significant difference in outcome is defined as at least a 10 %-points greater rate of abstinence or drinking in a controlled manner in treatment group 2 compared to group 1.

#### Secondary outcomes

- Proportion of total abstinence in each treatment group at 6-month follow-up
- Percentage of patients with abstinence or controlled use (maximum daily alcohol intake equivalent to BAC ≤ 0.05 %):
  1. In the last 7 days before regular end of treatment (end of MET and end of MET + CRA, respectively)
  2. In the last 30 days at 12 month follow-up
- Quality of life
- The proportion of clients completing the planned treatment

### Statistical analyses

Multiple logistic regression models will be used to analyse primary and secondary outcomes, and explanatory variables will include treatment, site, gender and age as well as other relevant demographic variables. The statistical analyses will be based on an “intent-to-treat”-approach, thus all patients randomized to treatment will be included. A multiple imputation strategy will be used in case of missing explanatory variables. The primary hypothesis of differences between treatment conditions will be tested one-sided since MET + CRA treatment is an extension of MET treatment. All other hypotheses will also be tested one-sided. In addition, we will perform a participation analysis where a logistic regression analysis is used to investigate how available demographics characteristics affect the probability that an individual will accept participation in the study.

The primary outcome is abstinence *or* controlled use as stated above. Information from the Form-90 is used to calculate the BAC based on the Widmark formula with an age adjustment [43].

#### Power calculation

The number of patients is based on a power calculation with an expectation that at least 50 % of patients in MET treatment and 60 % of patients in the MET + CRA will have a good clinical outcome six months after treatment initiation as defined above in primary outcome. Since MET + CRA is an extension of MET, we will employ 1-sided hypothesis testing. With a significance level of 5 % (one-sided) and a power of 90 % each treatment group, MET and MET + CRA, will require 423 patients. As we foresee a dropout rate at approximately 15 %, we will strive for 500 patients in each group.

### Quality assurance and fidelity monitoring

Several procedures are employed to ensure and document fidelity of the two study treatments.

*All therapists are trained in both treatments*. The therapists are assigned before randomisation and must therefore be able to deliver both. As 3 different countries and 16 sites participate, the training program has two steps: First, a minimum of 2 therapists from each sites participated in a “train-the-trainers” – conference in Denmark. At this 5-day conference the therapists were introduced to the therapies and trained extensively. These trained therapists subsequently train all other participating therapists locally at each site. Therapy sessions are digitally audio-recorded. Local supervisors at each site, experienced in the treatment and in supervision, continuously review randomly drawn sessions from each therapist and give feedback to therapists to ensure concordance with the treatment manuals. On-going supervision includes biweekly Skype meetings between all local supervisors and the overall supervisors on the treatment delivery in order to discuss and synchronize treatment delivery across sites, and secure treatment manual adherence.

*Treatment fidelity monitoring* is based on digital recordings of all sessions performed in the study. A random sample equivalent to 10 % of all sessions is drawn and scored by independent coders using the methodology of MITI 4 (Motivational Interviewing Treatment Integrity) [44] to measure fidelity to the style of the therapy. In addition, the coders complete a checklist of the content in the sessions.

*Data collection* is performed by research assistants (interviewers) not involved in the treatment and blinded to treatment assignment. Data collection is based on manualized procedures, and data are directly entered into the database through iPads or computers. The data forms are programmed with logical rules and allowed values, thus assisting the interviewer during the process. Data quality and completeness is continuously monitored. Twenty percent of the air samples are randomly selected for analysis to validate the self-reported data.

### Ethical approval

The study is approved by the ethical committee systems following the local rules and regulations for participating in scientific research project in all 3 countries: In Denmark by The Regional Scientific Ethical Committees for Southern Denmark, project-ID S-2013138; Germany, project-ID EK 389102013 (the ethics committee, Technische Universitäet, Dresden) and Ethical Board of the German Society of Psychology (DGPs, Reg.-No. EK-Antrag Pfeiffer-Gerschel / Bühringer 12/2013, Munich); and USA, New Mexico, project-ID University of New Mexico HRRC #13-580. The study is also registered in the Clinical Trials gov database: NCT02084173.

### Organisation, administration, and oversight

The *Principal Investigator (PI)* of each country together with the director of the RESCueH program (a research program consisting of five Danish alcohol treatment studies: *R*elay Study, *E*lderly Study, *S*elf-match Study, *Cue* Exposure Study, and *H*ealthy Lifestyle Study) forms the principal decision-making body of the study (*Steering Committee, SC*). They oversee all aspects of the design, execution, and publication of the study. The SC has designated *subcommittees* to develop and monitor aspects of the study. Each of these subcommittees refers directly to the director, which brings issues/recommendations to the SC’s attention for discussion and decision/approval. Logistically, fortnightly Skype meetings are held in which all PIs and the director, including relevant members of the staff, participate. The subcommittees also meet via Skype at regular intervals to discuss issues pertaining to their area of responsibility.

The *official study language* is English. Manuals and all other study material are originally written in English and only patient-related material is translated into Danish, German and Spanish.

To manage revisions of protocol and treatment manuals, as well as securing that all study sites have access to the same versions of the different instruments and questionnaires, Microsoft’s SharePoint is used. The SharePoint, which is supervised by the SC, is also used for storing minutes from the different Skype meetings, as well as all other decisions made so everybody in the study has instant access to all relevant information.

The SC organizes *data management* in collaboration with OPEN (Odense Patient data Explorative Network) [45], which provides the infrastructure for data entry and databases. All data entry and databases are based on REDCap™ (Research Electronic Data Capture), which is a secure web application for building and managing online data entry systems [46]. Thus all data collection is based on directly entering interview data into the databases through iPads or computers. The REDCap instrument assists the interviewer by build-in security warnings, e.g. alerts if a value not allowed is entered. OPEN and REDCap also provide “in real time” monitoring of for example recruitment rates, which are distributed to all participating staffs in weekly newsletters. The newsletters are also used as communication of decisions made by the SC.

Finally, the SC meets once a year with an international *Advisory board* consisting of Emeritus Distinguished Professor William R. Miller, New Mexico, USA, Emeritus Professor Gerard Schippers, The Netherlands, Dr. Gillian Tober, United Kingdom, as well as representatives from the Lundbeck Foundation, the University of Southern Denmark, the Region of Southern Denmark, and The Municipality of Odense. The Dean of the Faculty of Health Sciences, University of Southern Denmark is chairman of the advisory board.

### Pilot study

From January 2014 to April 2014 pilot testing all the instruments and the logistics involved in the study was conducted. A total of 89 patients participated, and based on the experience we added The Brief Symptom Inventory (BSI) to evaluate psychological and psychiatric problems in more detail [36]. No other major changes were found necessary.

## Expected results

This study will present knowledge about brief treatment of AUD for older subjects, including knowledge about the effects of a brief intervention and the effects of targeting treatment to specific needs. The comprehensive assessment program will also gain insight into the characteristics of older adults seeking treatment, which may contribute to further refinement of treatment and prevention of AUD among older adults. In addition, the interventions are manualized and being tested under “real world” conditions in three different countries, so they could be implemented relatively easily in western countries if proven effective. As we have aimed for as few exclusion criteria as possible we believe the study will show effectiveness and not solely efficacy. This will be very useful when planning future treatments both for therapists and for organisers of health care services.

## Discussion

### Challenges

As the study is conducted in different countries with different treatment cultures and organisation of the health care system it is not possible to define a “treatment as usual (TAU)” across all countries. Therefore we have decided not to compare any of the treatment groups with a control group based on TAU. Based on experience from daily clinical work we have decided that a meaningful outcome is a success rate of at least 50 % and, if extra effort (the extended CRA modules) is worth pursuing, this shall gain at least 10 % higher success rate.

In that line of thinking, we have also discussed a clinical relevant outcome. We aim for abstinence, but, as some patients prefer “controlled use”, and reduction of alcohol use can be a relevant factor in terms of harm reduction, we have decided to define a success as abstinence or controlled use (maximum daily alcohol intake equivalent to BAC ≤ 0.05 %) in the last 30 days at 6 months after start of treatment.

### Innovative aspects

The present study will gain new insights into older adults seeking treatment for their alcohol misuse. This has only been the focus of a small number of studies so far. Not only will new data be available, but simultaneously from different countries and cultures across the Atlantic Ocean. This will add to the generalizability of the results or provide information about the cross-cultural differences in treatment effects. If the treatment offered is successful the study will also show that a brief intervention is feasible and is easy to implement for this age group. In addition, as the design is an add-on design, the study will gain information about whether a very brief intervention focusing on motivation and planning for change is sufficient or including modules dedicated to help the patient execute these changes achieve better outcomes. In addition, information on patient characteristics that predict better results in both treatment conditions will help to improve assignment of brief interventions to elderly subjects with AUD. Further, as a module in the adapted CRA is dedicated to the needs of older adults this will provide knowledge about whether such a target is relevant or not. Finally, the study will provide data on the use of hair samples when assessing alcohol use.
